# Patient-reported outcomes from the randomized ALICE trial evaluating the addition of atezolizumab to anthracycline-based chemotherapy in metastatic triple-negative breast cancer

**DOI:** 10.1016/j.breast.2026.104704

**Published:** 2026-01-19

**Authors:** K.G. Svalheim, N.K. Andresen, C. Bjerre, B. Gilje, E.H. Jakobsen, R.S. Falk, B. Naume, S. Kaasa, J.A. Kyte

**Affiliations:** a Department of Clinical Cancer Research, Oslo University Hospital, Oslo, Norway; b Institute of Clinical Medicine, University of Oslo, Oslo, Norway; c Department of Cancer Immunology, Institute for Cancer Research, Oslo University Hospital, Norway; d Department of Oncology, Rigshospitalet, Copenhagen, Denmark; e Department of Hematology and Oncology, Stavanger University Hospital, Norway; f Department of Oncology, Sygehus Sønderjylland, Sønderborg, Denmark; g Department of Oncology, Oslo University Hospital, Oslo, Norway; h Oslo Centre for Biostatistics and Epidemiology, Oslo University Hospital, Oslo, Norway; i Faculty of Health Sciences, Oslo Metropolitan University, Oslo, Norway

**Keywords:** Triple-negative breast cancer, Immunotherapy, Patient reported outcomes, Anthracyclines, Metastatic, Predictive marker

## Abstract

**Background:**

The ALICE trial demonstrated that adding atezolizumab to anthracycline-based immunomodulatory chemotherapy improved progression-free survival (PFS) in patients with metastatic triple-negative breast cancer (mTNBC), including those with PD-L1–negative tumors. Here, we report the patient-reported outcome measures (PROMs).

**Methods:**

Patients were randomized to receive chemotherapy plus atezolizumab (atezo-chemo) or chemotherapy plus placebo (placebo-chemo). PROMs were collected at baseline and weeks 9, 17, 25, and 49 using the EORTC QLQ-C15-PAL, Chalder Fatigue Questionnaire (CFQ), and Numeric Rating Scale (NRS) for pain.

**Results:**

PROMs were available from 64 of 68 patients. At week 9, mean changes from baseline favored the atezo-chemo arm across all QLQ-C15-PAL domains, CFQ scores, and NRS pain intensity. Time-to-deterioration analyses also favored atezo-chemo, with statistically significant hazard ratios for global quality of life (QoL; HR 0.24), emotional functioning (HR 0.30), and pain (HR 0.20). Pain—a pre-specified cardinal symptom—improved in the atezo-chemo group at all time points. At 12 months, PROMs indicated sustained tolerability. Better baseline PROM scores were associated with improved PFS and overall survival, especially among patients treated with atezolizumab. Patients with >median QoL score at baseline recorded improved PFS when treated with atezolizumab (HR 0.25), while patients with ≤median QoL score did not (HR 1.02).

**Conclusions:**

Adding atezolizumab to the study-chemotherapy in mTNBC improves both PFS and patient-reported quality of life, emotional well-being and symptom control. These findings support continued development of this combination regimen and suggest that baseline quality of life may serve as a useful predictor of immunotherapy benefit.

**Trial registration:**

NCT03164993, May 24^th^ 2017; https://clinicaltrials.gov/ct2/show/record/NCT03164993.

## Highlights

• Atezolizumab added to chemotherapy improved all patient-reported outcomes in metastatic TNBC compared to chemotherapy alone.
• Time-to-deterioration data showed significant benefit of atezolizumab for quality of life, emotional functioning and pain.
• Pain, a cardinal symptom, was consistently reduced in the atezolizumab arm across all time points.
• The atezolizumab–chemotherapy combination was well tolerated over 12 months.
• Higher baseline quality of life predicted greater benefit from atezolizumab, suggesting its potential as a selection marker.

## 1 Introduction

Triple-negative breast cancer (TNBC) is an aggressive subtype with minimal expression of estrogen receptor, progesterone receptor and human epidermal growth factor receptor 2 (HER2) [1]. Immune checkpoint inhibitors (ICIs) have improved outcomes in several cancers [2] and show some efficacy in TNBC [3].

Atezolizumab, a PD-L1–blocking antibody, was approved for PD-L1–positive metastatic TNBC (mTNBC) in combination with nab-paclitaxel following the IMpassion130 trial [4,5]. However, IMpassion131 showed no benefit when combined with paclitaxel [6], and IMpassion132 found no efficacy in early relapsing mTNBC [7]. As a result, atezolizumab remains approved for mTNBC in Europe but not in the US, whereas pembrolizumab is approved in both regions based on KEYNOTE-355 data [8]. Recently, antibody-drug conjugates have expanded mTNBC treatment options [9]. Despite these advances, survival remains limited [10], emphasizing the importance of symptom relief and maintaining quality of life (QoL) [11,12].

The ALICE trial was the first placebo-controlled study to evaluate ICIs with anthracycline-based chemotherapy in metastatic breast cancer and the first to show benefit from adding ICI in PD-L1–negative mTNBC [13]. The rationale was to combine ICI with immunostimulatory chemotherapy [14]. All patients received pegylated liposomal doxorubicin (PLD) and low-dose cyclophosphamide, hypothesized to induce immunogenic cell death [[15], [16], [17]] and reduce regulatory T cells [18,19]. Patients were randomized to receive chemotherapy with either atezolizumab (atezo-chemo) or placebo (placebo-chemo). Previously reported results showed atezo-chemo was safe and improved progression-free survival (PFS) and time to QoL deterioration [13].

Patient-reported outcome measures (PROMs) systematically assess treatment effects from the patient's perspective [20] and support Shared Decision Making [20,21]. International guidelines recommend their use [22]. Here, we present PROMs from the ALICE trial. mTNBC typically causes rapid functional decline and worsening symptoms, often exacerbated by chemotherapy. We anticipated increasing symptoms in the placebo-chemo arm, while atezo-chemo patients might experience additional immunologic toxicity [23]. Prior ICI-chemo trials in TNBC found similar PROMs trajectories between arms [24,25].

Patient-reported outcomes (PROs) were secondary outcomes in ALICE [14]. We used the European Organization for Research and Treatment of Cancer Quality of Life Questionnaire Core 15 Palliative Care (EORTC QLQ-C15-PAL), a validated QoL tool for advanced cancer [26,27]. This is a shortened version of the EORTC-QLQ-30, where the number of items are reduced to decrease the burden to patients. The breast cancer-specific modules EORTC-QLQ-BR23 and BR45 were not used for the same reason. Additional questionnaires assessed pain (Numerical Rating Scale) and fatigue [28] (Chalder Fatigue Questionnaire [29]), key mTNBC symptoms. Pain is mainly cancer-related and was expected to mirror anti-tumor effect, while fatigue was expected to reflect both toxicity and tumor burden.

## 2 Patients and methods

### 2.1 Patients

The design and patient characteristics of the ALICE trial (NCT03164993) are described previously [13,14]. ALICE was a randomized, placebo-controlled phase IIb trial evaluating the efficacy of atezolizumab in addition to anthracycline-based chemotherapy in patients with mTNBC. Patients were randomized 3:2 to receive, in addition to chemotherapy (PLD 20 mg/m^2^ every 2nd week plus cyclophosphamide 50 mg/day every other 2 week cycle), either atezolizumab 840 mg i.v. (n = 40) or placebo (n = 28). Oslo University Hospital (OUH) was the trial sponsor. Patients were enrolled in five hospitals in Norway and Denmark (OUH, Stavanger University Hospital, St. Olavs Hospital, Vejle Hospital, Rigshospitalet).

### 2.2 PROM assessment

EORTC QLQ-C15-PAL consists of 15 questions, giving ten outcome scales: Quality of life, two functioning scales (emotional functioning, physical functioning), and seven symptom scales (fatigue, nausea/vomiting, pain, dyspnea, insomnia, appetite loss, constipation) [27]. Data were scored according to the EORTC QLQ-C15-PAL scoring manual [26]. Each item was rated “Not at all” to “Very much” by the patient, giving a score from one to four, except for the item “Quality of life”, which was rated “Very poor” to “Excellent” on a scale from one to seven. The scales were converted to a score of 0–100. Chalder Fatigue Questionnaire (CFQ) consists of 12 questions evaluating fatigue during the past 4 weeks, translating into one overall score (0–33 points), where a higher score means a higher level of fatigue [29]. NRS is an 11-point scale where patients score their pain intensity the last 24 h. All three questionnaires were completed prior to the study visits at baseline and on day 1 of cycle 5, 9, 13 and 25 (i.e. week 9, 17, 25 and 49). Each treatment cycle was 14 days.

Deterioration was defined as exceeding the minimal clinically important difference (MCID) thresholds in change from baseline for each of the scales. MCID in the EORTC QLQ-C15-PAL scales was defined as a change of ≥20 at the individual level and ≥10 at group level. MCID in the CFQ score was defined as a point change of ≥3 and ≥ 2 from baseline, at individual and group level, respectively. For the NRS, the MCID was defined as a change of ≥2 at individual level and ≥1 at group level.

### 2.3 Statistical analysis

Data processing and analysis were performed using Stata v.18.0 (StataCorp, USA/TX) and R v.4.3.2 (R Core Team, 2023). Patients missing baseline values were not included and those missing two or more consecutive assessments were censored for deterioration at their last assessment prior to this. No adjustment for differences in baseline values between the groups was performed.

The completion rate for each treatment arm on each PROM was calculated. The mean change in scores and the individual change in score of each outcome scale from baseline are described.

Time to deterioration (TTD) in scales from the EORTC QLQ-C15-PAL, CFQ and NRS were analyzed using Cox proportional hazard model. PRO values analyzed as continuous variables were scaled by dividing by the standard deviation. Results are presented as hazard ratios (HR) with 95 % confidence intervals (CI). TTD of select scales (QoL, emotional functioning, CFQ, NRS) were compared using the Kaplan-Meier method. *P* values were calculated using the log-rank method. Results were stratified by PD-L1 status. We furthermore investigated if QoL and emotional functioning at baseline (≤median versus > median scores) were associated with PFS and OS. All *P* values given are two-sided.

## 3 Results

### 3.1 Patient characteristics and compliance rate

**Fig. 1:**
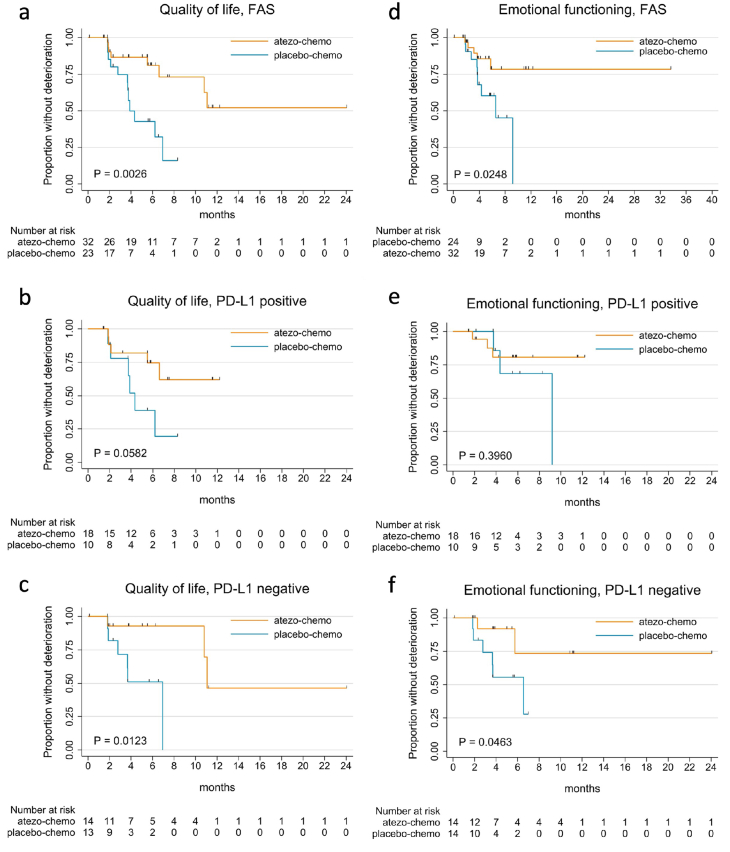
Kaplan-Meier plots for time to deterioration in quality of life for [a] all patients, [b] PD-L1-positive and [c] PD-L1-negative, and emotional functioning for [d] all patients, [e] PD-L1-positive, [f] PD-L1-negative. P-values were calculated with log-rank test.

Patient characteristics have been reported previously [13]. Overall survival (OS) data were updated as off 01.04.2025, giving a median follow up of 65.1 months. The only long-term survivor in the placebo-chemo arm received PD1/PD-L1 inhibitor therapy after end-of-treatment in ALICE. The EORTC QLQ-C15-PAL questionnaire was used at all study sites. Only patients treated at Norwegian centers responded to the CFQ and the NRS for pain intensity, as validated Danish versions were not available. At baseline, the completion rates for the PROMs were 64/68 patients (94 %) for EORTC QLQ-C15-PAL, 42/47 patients (89 %) for CFQ and 45/47 patients (96 %) for NRS. Supplementary (Suppl) Fig. 1 shows the proportion of patients remaining in the study arms at each time point. The compliance rate for each PROM remained above 76 % at all time points throughout the course of the study (Suppl. Table 1).

### 3.2 Baseline scores

Mean baseline scores for EORTC QLQ-C15-PAL, CFQ and NRS are shown in Suppl. Table 2. There was no statistically significant difference between the arms for EORTC QLQ-C15-PAL, but numerically lower scores were recorded on the QoL and functioning scales (physical functioning, emotional functioning) in the atezo-chemo arm. Also in the symptom scales, the placebo-chemo arm tended to score better (lower scores indicating less symptoms). CFQ scores at baseline showed more fatigue in the atezo-chemo arm (14.8 versus 11.8; p = 0.014). The atezo-chemo arm also tended to score higher than the placebo-chemo arm in the NRS (3.4 versus 2.3; p = 0.133), indicating higher baseline levels of pain intensity.

### 3.3 Time to deterioration

**Fig. 2:**
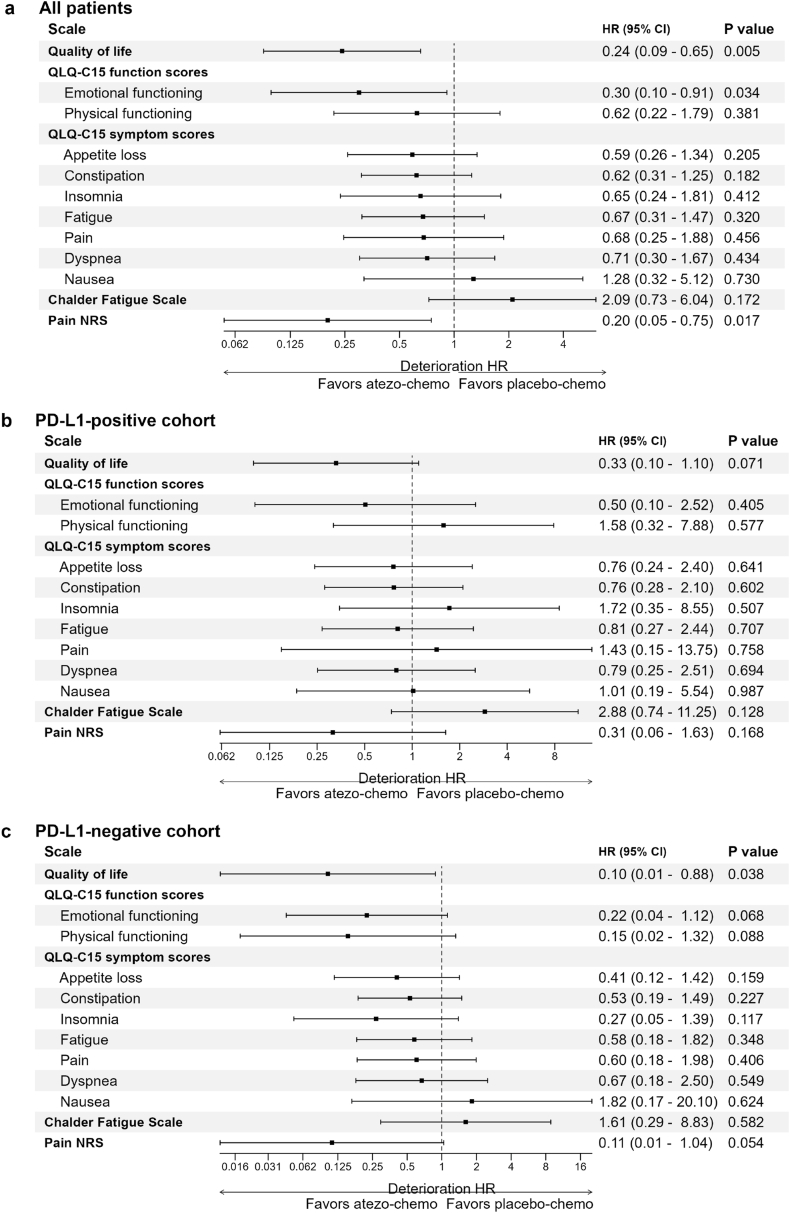
Time to deterioration (TTD) of PROMs in the atezo-chemo versus placebo-chemo arm. Forest plots with HRs for each PROM in [a] All patients, [b] PD-L1-positive cohort, [c] PD-L1-negative cohort. HRs, 95 % confidence intervals (CI) and p-values were calculated by cox regression analysis.

Due to the aggressive nature of mTNBC, coupled with side effects from therapy, the most patients experience rapidly worsening of symptoms and decreased QoL unless the therapy has clear anti-tumor activity. In ALICE, the time to deterioration (TTD) for most PROMs would be expected to be shorter for the atezo-chemo arm due to immune-toxicity, unless there was a considerable anti-tumor effect from atezolizumab. Fig. 1 (Fig. 1) shows Kaplan-Meier plots of TDD in QoL and emotional functioning (EF). For QoL, a statistically significant difference in favor of the atezo-chemo arm was observed for the total cohort (p = 0.0026), as well as in the PD-L1^negative^ subgroup (p = 0.012). A similar trend was seen for patients with PD-L1^positive^ disease (p = 0.058). These findings were supported by Cox regression analyses, indicating clinically important HRs between the arms (Fig. 2). The HRs were statistically significant for the total cohort (HR 0.24, p = 0.005) and the PD-L1^negative^ subgroup (HR 0.10, p = 0.038), with a trend for the PD-L1^positive^ subgroup (HR 0.33, p = 0.071).

The TTD for emotional functioning (EF) was also significantly longer in the atezo-chemo arm (Fig. 1d–f), with a HR of 0.30 (p = 0.034) in the total cohort (Fig. 2). Again, the recorded benefit appeared largest for patients with PD-L1^negative^ disease (HR 0.22, p = 0.068). The TTD for physical functioning and six out of seven symptom scales in EORTC QLQ-C15-PAL were also in favor of the atezo-arm in the total cohort, albeit not statistically significant (Fig. 2).

We selected pain as a cardinal tumor-related symptom, and hypothesized that the development of pain may mirror tumor burden, incorporating bone lesions that cannot be accurately measured by radiology. The NRS scores indicated that the time until development of increased pain intensity was significantly prolonged in the atezo-chemo arm (p = 0.017; Suppl. Fig. 2), with a HR of 0.20 in the total cohort (p = 0.017; Fig. 2). This advantage appeared to apply to patients with both PD-L1^positive^ (HR 0.31) and PD-L1^negative^ disease (HR 0.11). The recorded TTD for CFQ tended to be in favor of the placebo-chemo group, but this difference was not statistically significant, and the mean change in CFQ was in favor of the atezo-chemo arm (see below). No patients in the placebo-chemo arm remained in the trial beyond 9 months without deterioration in CFQ (Suppl. Fig. 2).

### 3.4 Changes in PROMs from baseline

**Fig. 3:**
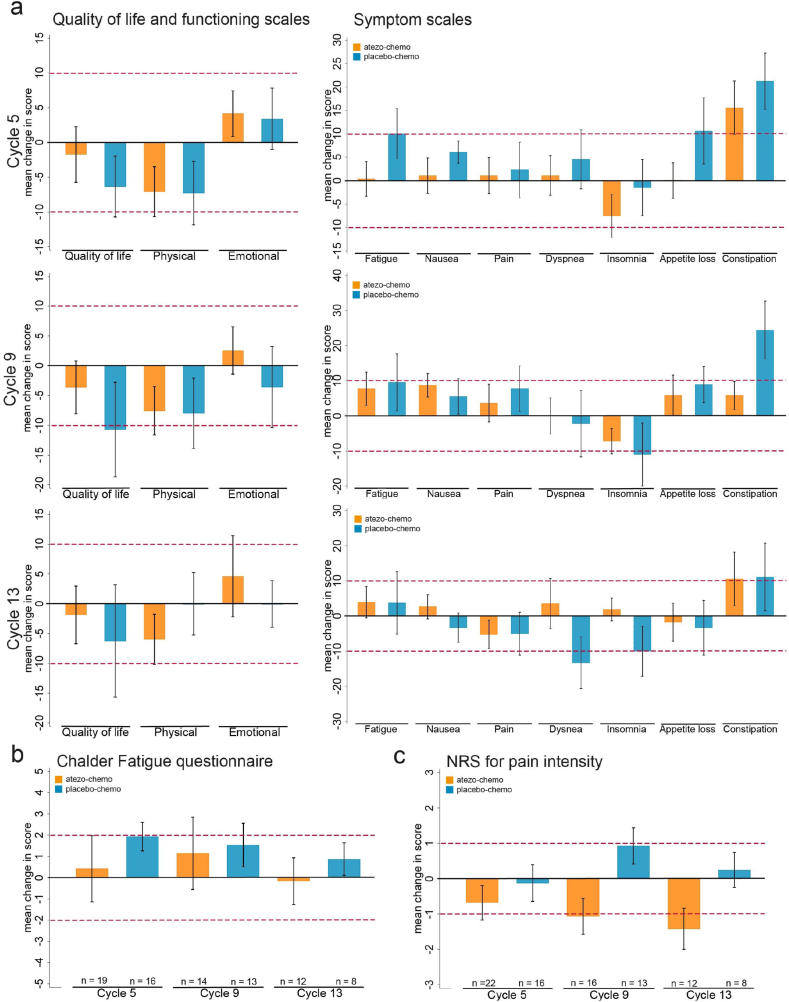
Changes in PROMs from baseline. (a) Mean change in EORTC QLQ-C15 PAL scales. Worsening is indicated by a score<0 for QoL and functional scales (left chart), but by a score>0 for symptom scales (right chart). (b) Mean change in Chalder Fatigue score. Worsening is indicated by a score>0. (c) Mean change in NRS pain intensity. Worsening is indicated by a score>0. Error bars represent SEM. Red dotted lines indicate the protocol-defined group-level thresholds for clinical importance. (For interpretation of the references to colour in this figure legend, the reader is referred to the Web version of this article.)

The mean changes at group level from baseline of EORTC QLQ-C15-PAL measures are shown in Fig. 3a, using the 10-point MCID threshold defined in the clinical protocol. In the placebo-chemo arm, a deterioration exceeding the MCID was found for QoL (cycle 9), fatigue (cycle 5), appetite loss (cycle 5) and constipation (cycles 5, 9, 13). In the atezo-chemo arm, an MCID was only observed for constipation (cycles 5, 13). By cycle 13, many patients in the placebo-chemo arm had ended study treatment, mostly due to disease progression. In the select group of placebo-chemo patients for whom cycle 13 data were obtained, improvements exceeding MCID were recorded for dyspnea and insomnia.

The atezo-chemo arm showed less worsening from baseline to cycle 5 in all scales compared to the placebo-chemo arm. Comparison between the arms at later time points was affected by a larger rate of patients ending study treatment in the placebo-chemo arm. The atezo-chemo arm exhibited less deterioration in most scales at cycle 9 as well. The distribution of individual change in each patient from baseline is shown in Suppl. Fig. 3.

Mean changes from baseline in the CFQ and NRS scores were in favor of the atezo-chemo arm at all time points. (Fig. 3b). Interestingly, the mean NRS pain score was lower in the atezo-chemo arm at cycle 5, 9 and 13 than at baseline, and the improvement reached MCID (Fig. 3c). This concurs with our pre-definition of assessing pain as a cardinal disease-related symptom. The distribution on patient level is shown in Suppl. Fig. 4.

### 3.5 PRO results in patients receiving long-term treatment

We further investigated PRO data collected from patients continuing treatment after 12 months. This would inform on the tolerability of long-term study treatment in this patient population, as well as disease-related symptoms. EORTC QLQ-C15-PAL data were obtained at cycle 25 (week 49) from eight out of ten patients that were still on treatment (Suppl. Fig. 5). Only one of these patients received placebo-chemo. In the atezo-chemo arm there was no MCID from baseline at group level in any of the QLC-C15-PAL functional scales (QoL, physical, emotional). In the symptom scales, a borderline MCID improvement of −9.5 points was recorded for pain, whereas the other six symptom scales did not reach a MCID. The 12-month data thus suggest that continued atezo-chemo combination treatment was well tolerated over time.

### 3.6 Prognostic value of PROs

**Fig. 4:**
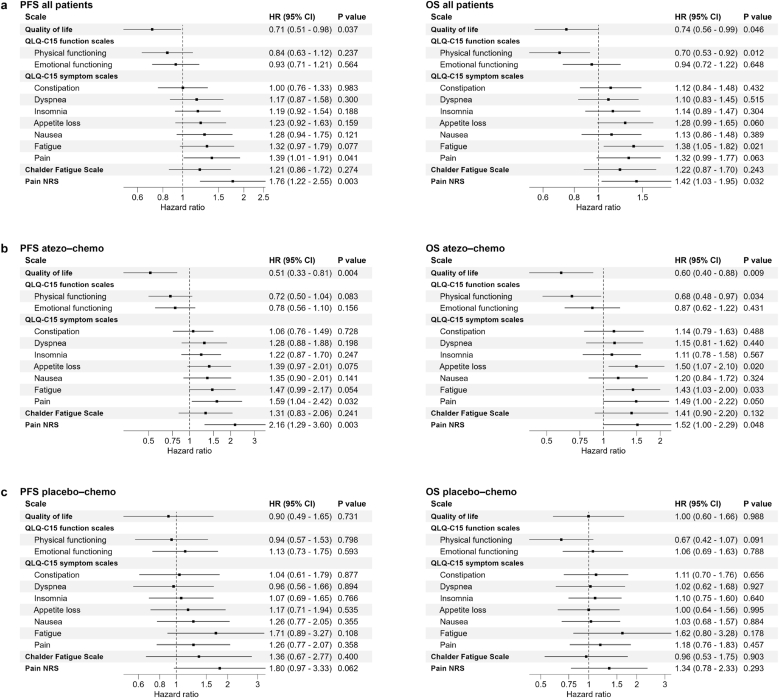
Prognostic value of baseline PROs. Forest plots with hazard ratios for PFS and OS for each PRO. (a) Data for all patients. Patients reporting better pre-treatment QoL, physical and emotional functioning had longer PFS/OS (HR < 1). For symptom scales, CFS and NRS, a HR < 1 indicate that patients with more baseline symptoms had shorter PFS/OS. (b) Data for atezo-chemo arm. (c) Data for placebo-chemo arm. HRs, 95 % confidence intervals (CI) and p-values were calculated by cox regression analysis. PRO values were analyzed as continuous variables and scaled by dividing by the standard deviation.

We furthermore investigated if PROs at baseline were associated with PFS and OS, analyzing both arms together. Fig. 4 shows the PFS and OS hazard ratios for each PROM. For the QoL and functioning scales in EORTC QLQ-C15-PAL, as well as 6/7 symptom scales, patients with a better score went on to experience improved PFS (Fig. 4a). The same applied for the CFQ and NRS pain score. The observed PFS associations were statistically significant for QoL (HR 0.71), Pain (HR 1.39) and NRS (HR 1.76). For OS, the associations reached statistical significance for QoL, physical functioning, fatigue, and NRS (Fig. 4a).

### 3.7 Predictive value of PROs for the benefit of atezolizumab

**Fig. 5:**
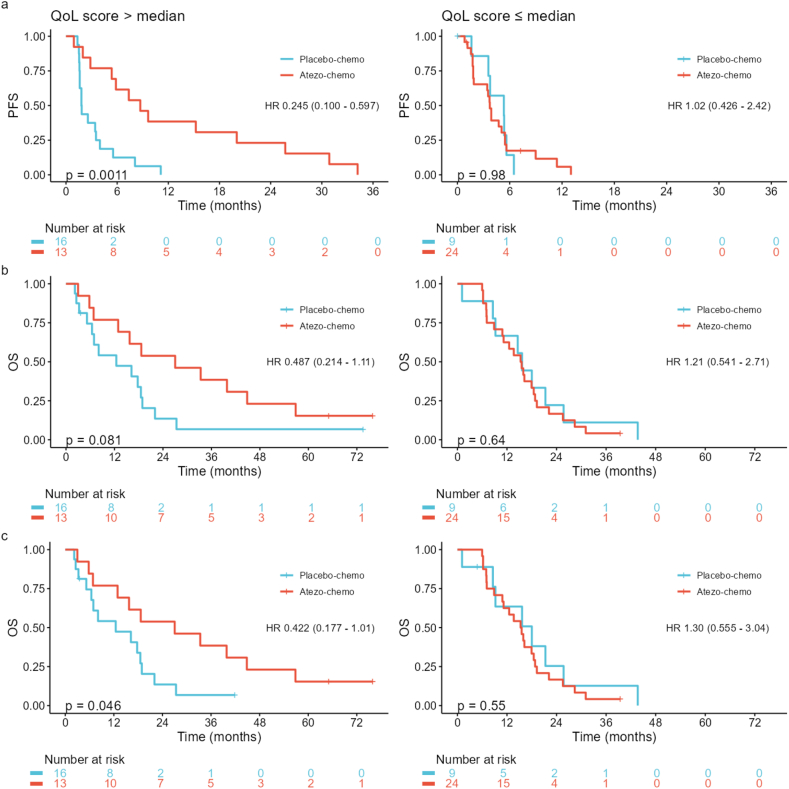
Predictive value of QoL score for benefit of atezolizumab. Kaplan-Meier plots for PFS (a) and OS (b–c), in the atezo-chemo compared to placebo-chemo arm, in patients with high (>median) or low (≤median) QoL score. Two patients in the placebo-chemo arm started therapy with PD1/PD-L1 checkpoint inhibitors after end-of-treatment in the ALICE trial. (b) OS, without censoring for any post-study therapy. (c) OS, with censoring at start of post-study PD1/PD-L1 checkpoint inhibitor therapy. Median QoL score was 66.7. P-values calculated with log-rank test. HRs were calculated by cox regression analysis.

The association between better baseline scores and improved PFS/OS was most prominent in patients receiving atezolizumab, across most PROs (Fig. 4b and c). For the QoL scale, a significant association was observed in the atezo-chemo arm for both PFS (HR 0.51; p = 0.004) and OS (HR 0.60; p = 0.009). By contrast, there was no such association in the placebo-chemo arm (PFS HR 0.90; OS HR.1.0) (Fig. 4c). Based on this, we investigated if baseline QoL was predictive of benefit from atezolizumab. The analysis showed that patients with >median QoL score had a strong PFS benefit from atezolizumab (p = 0.001; HR 0.25), whereas patients with ≤median QoL had no benefit (HR = 1.02) (Fig. 5a). For patients with >median QoL, there was also an evident indication of OS benefit from atezolizumab, while no such tendency was observed in the group with ≤median QoL (Fig. 5b and c). The results suggest that QoL may be a selection marker for benefit from atezolizumab.

Emotional distress has been associated with poor response to ICI in melanoma and non-small-cell lung cancer (NSCLC) [30,31]. We investigated if this also applied to the ALICE cohort and found that patients with high emotional functioning (EF) had a larger atezolizumab benefit, both measured by PFS (HR 0.30 versus HR 0.70) and OS (HR 0.49 versus 0.89) (Suppl. Fig. 6). There was though also a tendency of atezolizumab benefit in patients with low EF, so that QoL appeared to be a more suitable selection marker.

## 4 Discussion

ALICE trial was a small randomized trial, but demonstrated that the addition of atezolizumab to chemotherapy significantly improved progression-free survival (PFS; HR 0.57), which was the primary endpoint [13]. Patients with mTNBC are not cured, and it is important to be cautious with escalating therapy based on PFS, as this may improve even when the QoL decreases due to toxicity. Therefore, it is noteworthy that patients in the ALICE atezo-chemo arm experienced favorable PROs compared to placebo-chemo. This observation on group level was consistent across most measures, with a statistically significant difference in TTD for QoL, emotional functioning (EF) and the NRS pain scale. The hazard ratios (HRs) for these measures were sizeable (HR 0.20–0.30). These data from the patient's perspective are in good agreement with the previously reported findings from radiological assessments and safety monitoring, indicating that the combination therapy improved tumor control and was well tolerated. Moreover, by adding PRO data into the equation it was possible to identify patients with poor QoL as having lower chance of a positive effects from the addition of immunotherapy.

When intensifying therapy, the symptomatic benefit is expected to result from improved tumor control and to be countered by increased toxicity [23]. We still observed a larger advantage in key PROs, than for PFS or objective tumor response [13]. Compared to PFS, the TTD plots for QoL, EF, and pain (NRS) suggested that a larger fraction of patients experienced benefit. The results thus highlight that PROMs may reveal effects not captured by radiological assessments and criteria, which define PFS. In our study, most patients had bone lesions and other metastases not measurable by RECIST.

It is noteworthy that the atezo-chemo group in ALICE reported improved PROs, compared to the placebo-arm. In mTNBC, the IMpassion130 and Keynote-355 trials showed no difference in PROMs between the immune-chemo and chemotherapy-only arms [24,25]. This is still considered to indicate that immunotherapy was well tolerated and, along with PFS and OS data, support the use these immune-chemo combinations. In neoadjuvant treatment of early breast cancer, IMpassion031 indicated that the addition of atezolizumab to chemotherapy did not significantly impact PROs [32].

PD-L1^negative^ mTNBC carries the poorest prognosis among breast cancer subgroups and has no option for immunotherapy. Therefore, it is important that the ALICE PROs indicate a clinically meaningful effect against PD-L1^negative^ mTNBC. Across most PROs, the atezolizumab benefit was strongest for patients with PD-L1^negative^ disease. This observation is in line with the PFS data and the secondary efficacy outcomes (tumor response, clinical benefit rate, overall survival), all indicating a larger effect against PD-L1^negative^ disease. ALICE was the first trial to show efficacy of adding immunotherapy to chemotherapy in PD-L1^negative^ mTNBC. In IMpassion130, no effect was found against PD-L1^negative^ disease [5]. Importantly, the same method was applied for determining PD-L1 status in ALICE and IMpassion130 [5,13]. While IMpassion130 used nab-paclitaxel as the chemotherapy backbone, ALICE employed pegylated liposomal doxorubicin, based on data suggesting that anthracyclines may be more potent inducers of immunogenic cell death [[15], [16], [17]]. The findings are thus consistent with the hypothesis of using anthracyclines to sensitize PD-L1^negative^ tumors to PD-L1 blockade. Data from the TONIC trial also supported this strategy in mTNBC [33]. In ALICE, we used the pegylated liposomal formulation of doxorubicin to avoid steroids and deep lymphopenia [34], and metronomic cyclophosphamide to reduce Tregs [18,19]. Flow cytometry measurements confirmed a selective decrease in Tregs both in ALICE [13] and in another breast cancer trial, ICON [35], where we applied the same chemotherapy. There is thus a rationale for explaining why atezolizumab appeared effective against PD-L1^negative^ disease in ALICE, even if it was not in IMpassion130 [36]. This finding supports a potential for immunomodulatory chemotherapies in general and is of interest beyond mTNBC and the ALICE combination. The mechanism of effect against PD-L1^negative^ disease should though be further investigated. Regardless of mechanism, the PRO data strengthen the finding from PFS and other previously reported outcomes, indicating that atezolizumab provided a clinically meaningful benefit.

Immunotherapy gives only moderate response rates in most cancers, but is often noted for a proportion of long-term responders [37,38]. In the ALICE trial, there was a similar pattern. The majority of patients had a short PFS, but all five patients with PFS beyond the pre-defined threshold of 15 months belonged to the atezolizumab group. For such patients, a common question is whether to continue treatment. The ALICE chemotherapy was selected to allow for long-term treatment by using PLD rather than traditional anthracyclines, where cumulative heart toxicity makes this impossible. As reported above, PROs after 12 months treatment showed a favorable development in symptoms, functioning, and QoL. Along with the safety data, the 12-month PROMs suggest that the combination regimen is well tolerated and represents a meaningful option for patients that may live for many years with disease.

The comparison between arms of changes from baseline is most informative at cycle 5 (week 9), before patients discontinued. Moreover, the patients filled in the forms at cycle 5 before learning the result of their first radiological evaluation, which means the readout was not influenced by this knowledge. Patients reported less pain in the atezo-chemo arm at all post-baseline time points, both in the NRS and EORTC QLQ-C15-PAL pain scales. This is of high clinical interest, as pain is a major concern for mTNBC patients. Completion rates for the PROMs were high at baseline (89–96 %) and remained above 76 % at all time points in both arms. It is possible that patients not answering the forms differed in their symptom burdens, thus comparison between treatment arms need to be interpreted with caution. For this comparison, it is though important that the trial was placebo-controlled and that few patients experienced adverse events indicating which arm they belonged to.

The mean baseline scores were generally worse in the atezo-chemo arm. No adjustments for differences in baseline values between the groups was performed in the statistical analyses, as we did not expect these differences to affect the TTD. It is unclear how this imbalance may have influenced the results. On the one hand, patients in the atezo-arm had more room for improvement in their PROs than the placebo-chemo arm. On the other hand, patients in poorer condition at baseline often fare worse in clinical trials. Poorer baseline scores was a negative prognostic factor for both PFS and OS in this trial, and one may speculate that the imbalance in baseline PRO scores may have led to underestimation of the OS benefit for the atezo-chemo arm. However, it is clear that a new trial would be needed to establish if the atezo-chemo combination provides a survival benefit.

The fact that many mTNBC patients show no response to ICI, while some experience long term benefit, highlights the need to better select patients [39]. In translational studies on material from ALICE, we have found that an immune gene signature in tumor, Treg levels and lymphocyte signatures in blood and a diverse gut microbiota were associated with atezolizumab benefit [13,40,41]. PROMs represent a different source of information that could complement biological biomarkers. In a previous melanoma ICI trial, we found PROMS to be prognostic [42]. In ALICE, we find that better PROMs at baseline were associated with a favorable clinical outcome, and that this applied both to OS, PFS and tumor response rate. Moreover, this association was stronger in the atezo-chemo arm, and the benefit from atezolizumab was confined to patients with above median score in the QoL scale at baseline. The latter point suggests that PROMs may be used for selecting patients for ICI therapy. Mechanistically, it is not known why baseline PROMs would be associated with effect of immunotherapy. However, one may speculate that this is related to the common observation that physical and psychological distress renders patients as well as healthy individuals more susceptible to infections. Several studies in animal models have indicated that durable stress impair the efficacy of PD-1/PD-L1 inhibitors [[43], [44], [45]]. In the ALICE patients, it is moreover possible that pre-existing immune activation correlated with a better QoL. Furthermore, better QoL at baseline may reflect less disease burden.

We found that high emotional functioning (EF) was also associated with larger benefit from atezolizumab. As mentioned, this observation is in line with studies from NSCLC [30] and melanoma [31]. In the NSCLC study, patients with increased emotional distress moreover had higher cortisol levels [30]. It is well known that emotional and physical stress activates the hypothalamic–pituitary–adrenal axis and the sympathetic nervous system, which leads to the secretion of stress hormones, including glucocorticoids. Furthermore, increased cortisol levels dampens the immune system, through effects of lymphocytes and neutrophils, as well as on antigen presentation [46]. Regardless of mechanism, these observed associations between QoL/EF and treatment response in mTNBC (ALICE), NSCLC and melanoma are of interest. Importantly, the findings indicate that these PROMS are not merely prognostic, but predictive of ICI efficacy. Patients with ≤median QoL score in ALICE had no benefit from atezolizumab. If the EORTC-QLQ-C15-PAL QoL scale or another specific score could be validated as a selection marker, this could be incorporated into clinical decision making.

The study has certain limitations. First, it is a relatively small randomized trial. Second, this study reports on secondary outcomes and are descriptive in nature. Third, the proportion of patients remaining in the study is higher in the atezo-chemo arm than in the placebo-chemo arm. Fourth, any comparisons between trials should be considered with caution. IMpassion130 and Keynote-355 used the EORTC-QLQ-C30, while we used the shortened version QLQ-C15-PAL, developed based on item response theory [47]. QLQ-C15-PAL has to our knowledge not been used in trials evaluating breast cancer immunotherapy. However, it has been reported that QLQ-C15-PAL scores are directly comparable with scores derived from QLQ-C30, and that this facilitates interpreting results in relation to published literature using QLQ-C30 [27].

We conclude that the patients experienced a clinically meaningful improvement from the addition of atezolizumab to anthracycline-based chemotherapy in mTNBC, including significantly better quality of life, emotional functioning and pain control. The benefit from atezolizumab was particularly prominent for patients with PD-L1^negative^ disease. Of note, the observed advantage in PROs was larger than expected based on PFS data, suggesting low toxicity combined with meaningful symptomatic improvement beyond what is captured by radiological assessments. Long-term combination treatment was well tolerated. Pre-treatment QoL and EF may be useful as indicators for better selection of patients to be treated with atezolizumab. These findings emphasize the importance of integrating PROs in clinical trials as well as in routine clinical practice. Along with the previously reported efficacy and safety data from ALICE, the PROs support using anthracycline-based regimens to sensitize tumors for CPI, particularly in PD-L1^negative^ patients. Further studies are needed to confirm the findings and optimize patient selection.

## CRediT authorship contribution statement

**K.G. Svalheim:** Writing – review & editing, Writing – original draft, Investigation, Formal analysis. **N.K. Andresen:** Writing – review & editing, Investigation, Data curation. **C. Bjerre:** Writing – review & editing, Investigation. **B. Gilje:** Writing – review & editing, Investigation. **E.H. Jakobsen:** Writing – review & editing, Investigation. **R.S. Falk:** Formal analysis, Methodology, Writing – review & editing. **B. Naume:** Writing – review & editing, Investigation, Funding acquisition. **S. Kaasa:** Writing – review & editing, Methodology, Conceptualization. **J.A. Kyte:** Writing – review & editing, Writing – original draft, Supervision, Methodology, Investigation, Funding acquisition, Formal analysis, Conceptualization.

## Patient consent for publication

NA.

## Data availability statement

Any request for raw or analyzed data will be reviewed by the study team, and a response can be expected within 14 days. Requests should be made to the corresponding author (jonky@ous-hf.no).

## Ethics approval

The study was approved by the Norwegian Medical Agency (ID: 16/11993), the Danish Medicines Agency (ID: 2018051636), Regional Committee for Medical Research Ethics South-East Norway (EC ID: 14195), the Research Ethics Committee in Denmark (EC ID: H-18018750), and institutional review boards. Trial conduct followed the ethical principles of the World Medical Association's Declaration of Helsinki (1964), the guidelines of Good Clinical Practice and the CONSORT 2010 guidelines. All patients provided written informed consent.

## Funding

The study was supported by grants from the Norwegian Health Region South-East (grants 2017100 to JAK and 2017122 to BN), the 10.13039/100008730Norwegian Cancer Society/Norwegian Breast Cancer Society (grants 182632 ***and*** 214972 to JAK) and the 10.13039/501100005416Norwegian Research Council (student grant to KGS/JAK). Roche supported the ALICE trial with free drug (atezolizumab), free SP142 kits, and a funding contribution, but 10.13039/100031365Oslo University Hospital was the trial Sponsor. Roche reviewed the clinical protocol, without responsibility for the study design, and reviewed the present manuscript before submission. None of the funders had any role in the design of the present study, the data collection/analysis/interpretation, the writing of the report or in the decision to submit the article for publication.

## Declaration of competing interest

The authors declare the following financial interests/personal relationships which may be considered as potential competing interests: J.A.K. has the last 5 years received research support from Bristol Myers Squibb, F. Hoffmann-La Roche, NanoString, and NEC Oncoimmunity and has previously received advisory board/lecture honoraria from pharmaceutical companies, including Roche. B.G. has received honoraria for advisory boards from Astra Zeneca, Eli Lilly, Gilead, Daiichi Sankyo, Roche, and Pierre Fabre. C.B. has received travel grants or advisory board/consultant honoraria from Novartis, Eli Lilly, Pfizer, Astra Zeneca, Gilead Sciences, MSD and Daichii Sankyo. All other authors declare no competing interests.
