# Supplementary material for: Patient-reported outcomes from the randomized ALICE trial evaluating the addition of atezolizumab to anthracycline-based chemotherapy in metastatic triple-negative breast cancer
Source: Breast. 2026 Jan 19;86:104704. doi: 10.1016/j.breast.2026.104704 (PMC12860362; doi:10.1016/j.breast.2026.104704)
Supplement: Multimedia component 1 [file mmc1.pdf]

Supplementary Figures og Tables

Patient-Reported Outcomes from the ALICE trial evaluating addition of atezolizumab to anthracycline-based chemotherapy in metastatic triple-negative breast cancer

Authors**:** KG Svalheim, NK Andresen, C Bjerre, B Gilje, EH Jakobsen, RS Falk, B Naume, S Kaasa, JA Kyte

Suppl. Table 1

Completion rate throughout the study

	baseline	cycle 5	cycle 9	cycle 13	cycle 25
**EORTC QLQ-C15-PAL**	64 (94.1%)	56 (88.9%)	38 (86.4%)	30 (90.9%)	8 (80%)
Placebo-chemo	26 (92.9%)	24 (96%)	15 (93.8%)	10 (90.9%)	1 (100%)
Atezo-chemo	38 (95%)	32 (84.2%)	23 (82.1%)	20 (90.9%)	7 (77.8%)
**Chalder Fatigue questionnaire***	42 (89.4%)	39 (88.6%)	29 (85.3%)	22 (88%)	6 (85.7%)
Placebo-chemo	17 (94.4%)	17 (100%)	13 (100%)	8 (88.9%)	1 (100%)
Atezo-chemo	25 (86.2%)	22 (81.5%)	16 (76.2%)	14 (87.5%)	5 (83.3%)
**NRS pain intensity***	45 (95.7%)	39 (88.6%)	29 (85.3%)	21 (84%)	6 (85.7%)
Placebo-chemo	18 (100%)	16 (94.1%)	13 (100%)	8 (88.9%)	1 (100%)
Atezo-chemo	27 (93.1%)	23 (85.2%)	16 (76.2%)	13 (81.3%)	5 (83.3%)

Supplementary Table 1: Completion rate of the questionnaires. The percentage is calculated based on the patients remaining in the study at the different time points. *Chalder Fatigue questionnaire and NRS pain intensity were only answered at Norwegian centers. There were 47 patients at the Norwegian centers at baseline.

Suppl Table 2

Baseline scores

	Placebo-chemo			Atezo-chemo			P-value
	n	mean	95% CI	n	mean	95% CI	
**EORTC QLQ-C15 PAL**							
Functioning scales							
Quality of life	25	76.0	68.3 - 83.7	37	66.7	59.5 - 73.8	0.075
Physical functioning	26	76.9	68.7 - 85.1	38	71.2	64.9 - 77.5	0.266
Emotional functioning	26	85.3	78.9 - 91.7	36	77.3	71.0 - 83.6	0.076
Symptom scales							
Fatigue	25	26.2	17.6 - 34.8	37	34.8	27.2 - 42.4	0.130
Nausea and vomiting	26	5.8	0.1 - 11.5	38	7.0	1.4 - 12.7	0.751
Pain	26	25.0	15.6 - 34.4	38	30.7	22.5 - 38.9	0.353
Dyspnea	26	19.2	8.3 - 30.1	38	21.9	11.65 - 32.2	0.714
Insomnia	26	29.5	17.9 - 41.1	38	22.8	15.1 - 30.5	0.331
Appetite loss	26	7.7	0 - 15.6	38	16.7	7.9 - 25.4	0.125
Constipation	26	11.5	4.0 - 19.1	37	12.6	6.6 - 18.7	0.821
**Chalder Fatigue Scale**	17	11.8	10.2 - 13.3	25	14.8	12.9 - 16.7	0.014
**Pain NRS**	18	2.3	1.1 - 3.6	27	3.4	2.6 - 4.3	0.133

Suppl Tab. 2: Mean baseline scores for all PROs. n, number of patients. 95% CI, 95% confidence interval. P-values are calculated by two-sample t-tests with unequal variances.

Suppl. Fig. 1

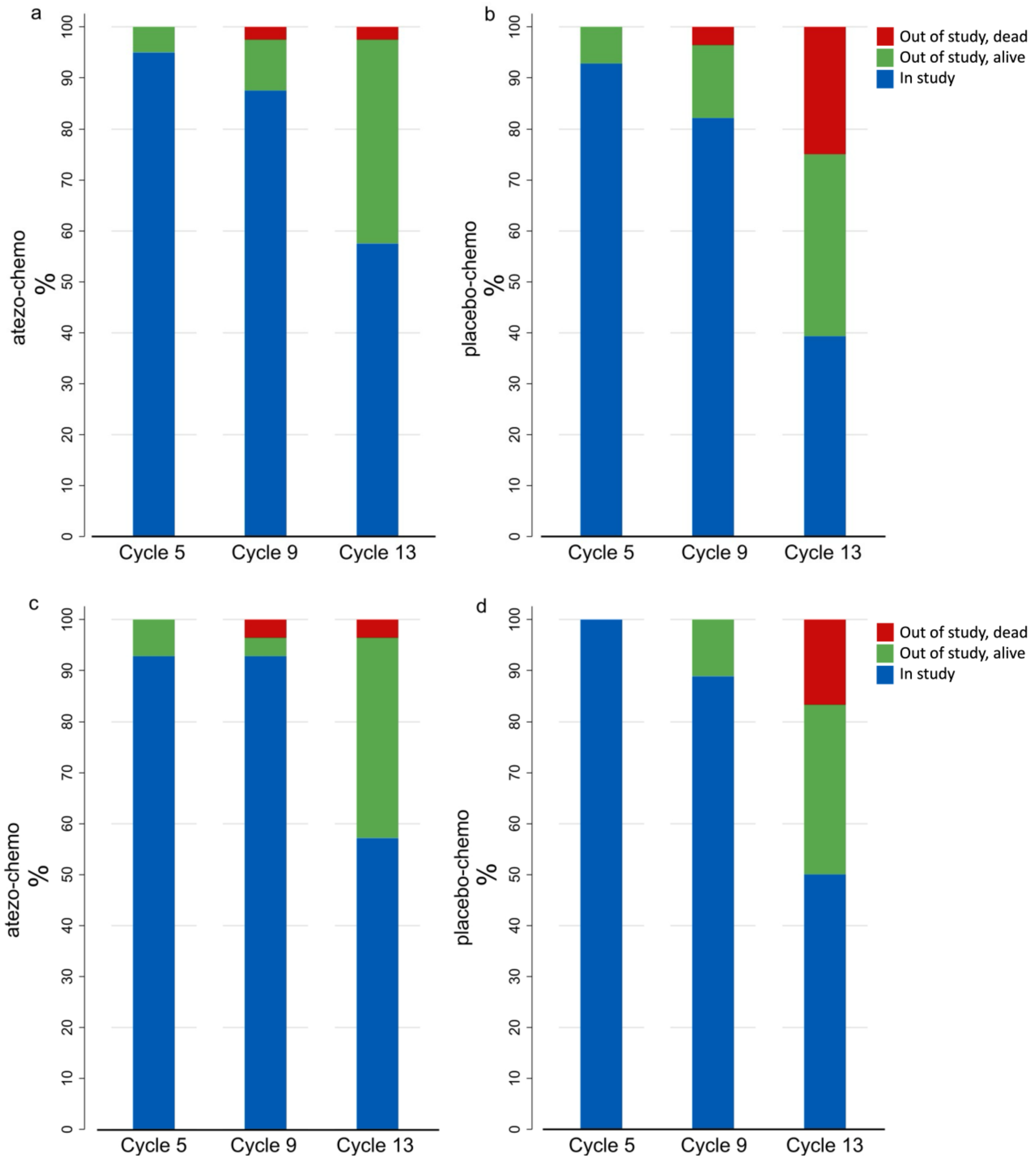

Suppl. Fig. 1: Bar plot showing the proportion of patients remaining in the study at each time point, and the proportion of patients alive. Data for EORTC-QLC-C15-PAL represent the proportion of FAS (n=68) and are shown in [a] (atezo-chemo arm) and [b] (placebo-chemo arm). Data for the Chalder Fatigue/ NRS pain intensity questionnaire, which were only answered at Norwegian centers (n=47), are shown in [c] (atezo-chemo arm) and [d] (placebo-chemo arm).

Suppl. Fig. 2

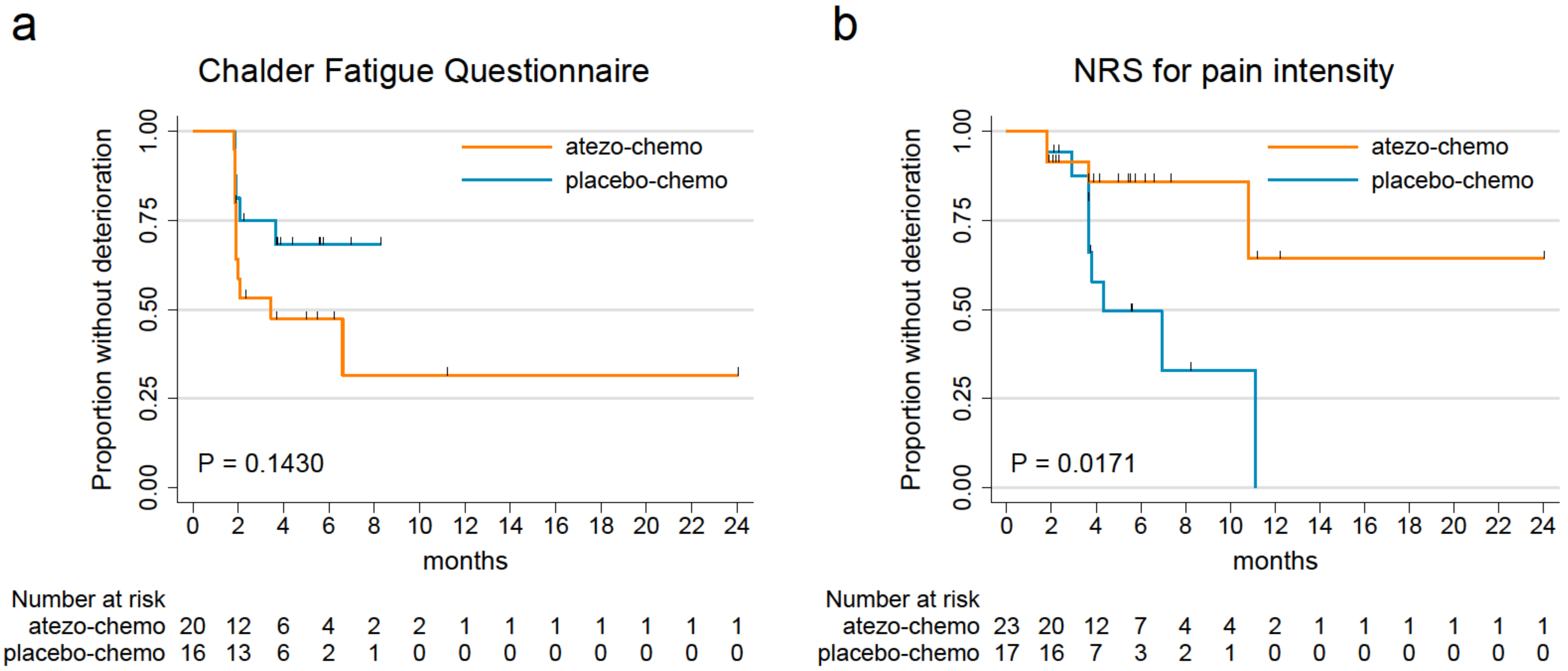

Suppl. Fig. 2: Kaplan-Meier plots for time to deterioration in [a] Chalder Fatigue score, [b] the NRS for pain intensity. P-values were calculated with log-rank test.

Suppl. Fig. 3

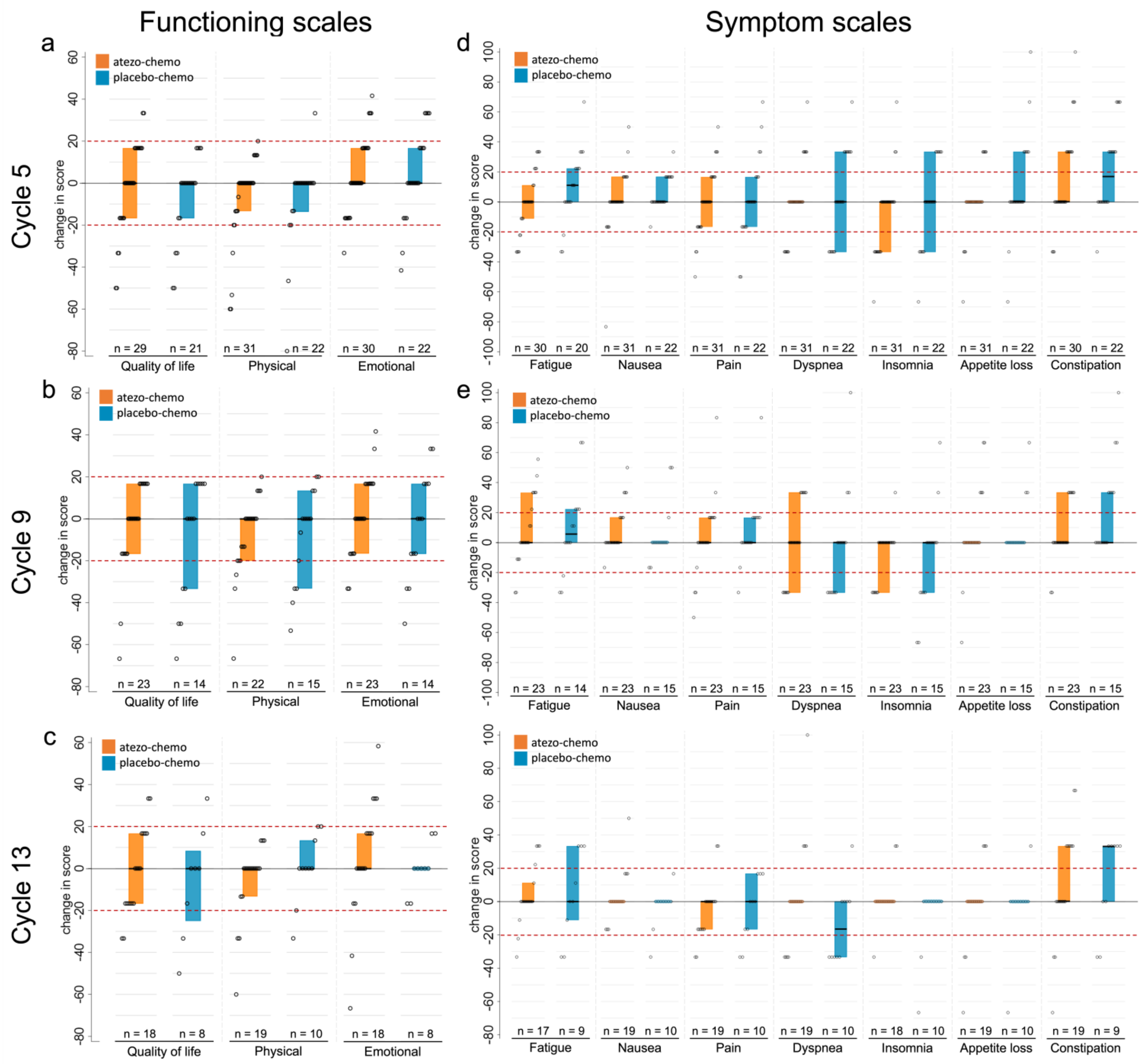

Suppl. Fig. 3: Scatter-box-plot for individual change in each patient in EORTC QLQ-C15-PAL functioning and symptom scales from baseline to [a, d] cycle 5, [b, e] cycle 9, [c, f] cycle 13. Red dotted lines show individual-level threshold for change of clinical importance. Median change is marked with a black line (not visible if median equals zero). Worsening is indicated by a score<0 for QoL and functional scales (left chart), but by a score>0 for symptom scales (right chart).

Suppl. Fig. 4

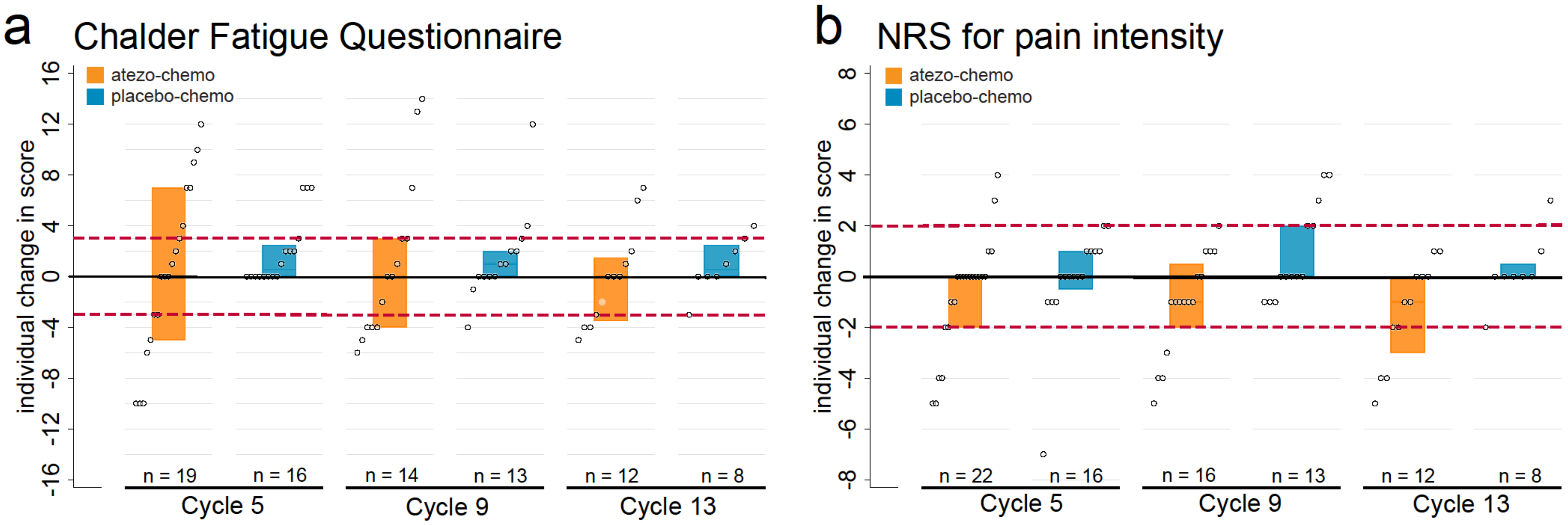

Suppl. Fig. 4: Scatter-box-plot for individual change in [a] Chalder Fatigue score and [b] NRS pain intensity from baseline. Red dotted lines show the individual-level threshold for change of clinical importance. Median change is marked with a black line (not visible if median equals zero).

Suppl. Fig. 5

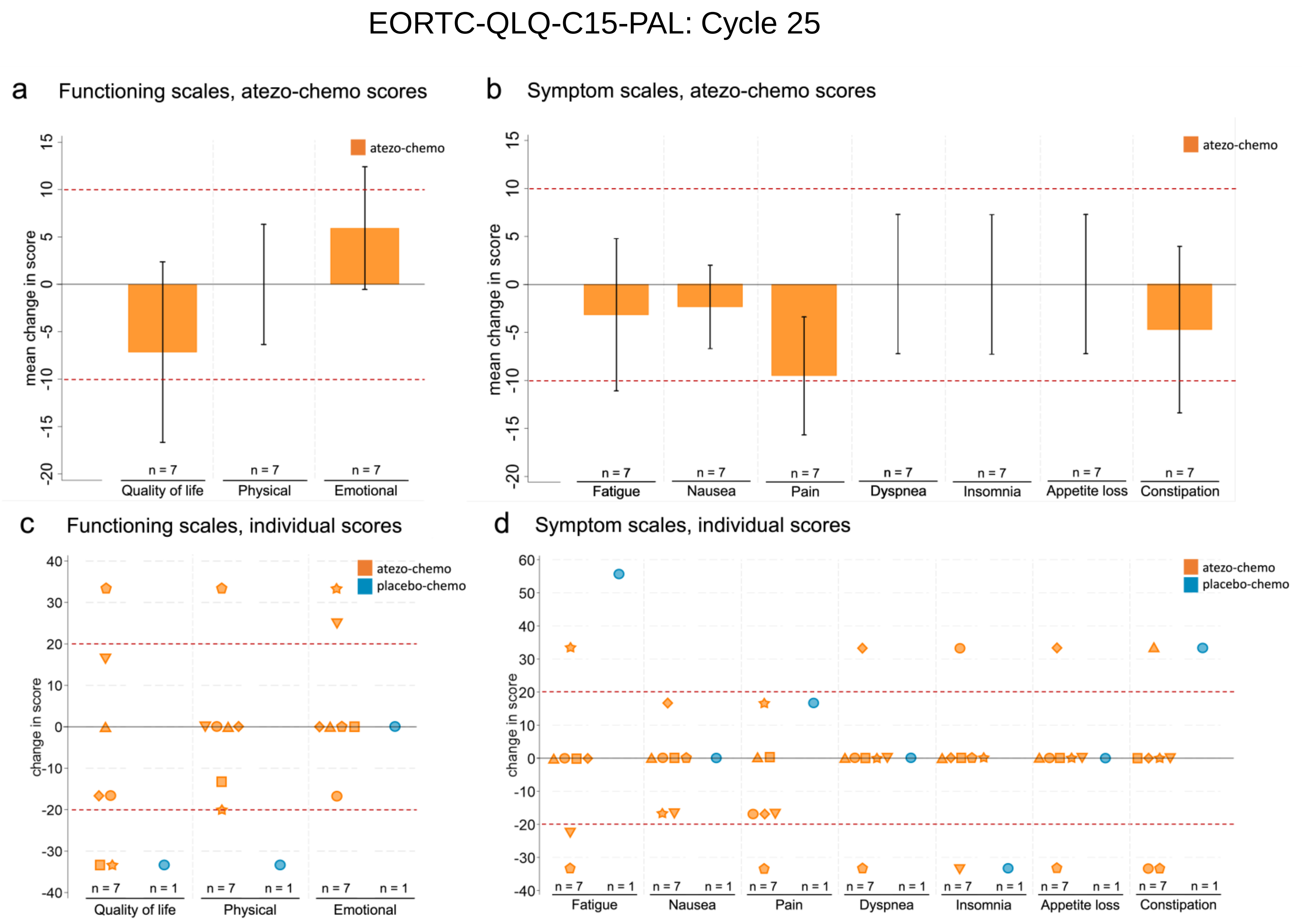

Suppl. Fig. 5: Development in QLQ-C15-PAL scales upon long term treatment. Changes in scores from baseline to cycle 25 (week 49). Data were obtained at cycle 25 from eight of ten patients that were still on treatment (7 in atezo-chemo arm, 1 in placebo-chemo arm). a) Group level changes in functioning scales. b) Group level changes in symptom scales. c) Individual changes in functioning scales. d) Individual changes in symptom scales. In c) and d) each patient is represented by different shapes. Worsening is indicated by a score<0 for QoL and functional scales (a and c), but by a score>0 for symptom scales (b and d).

Suppl. Fig. 6

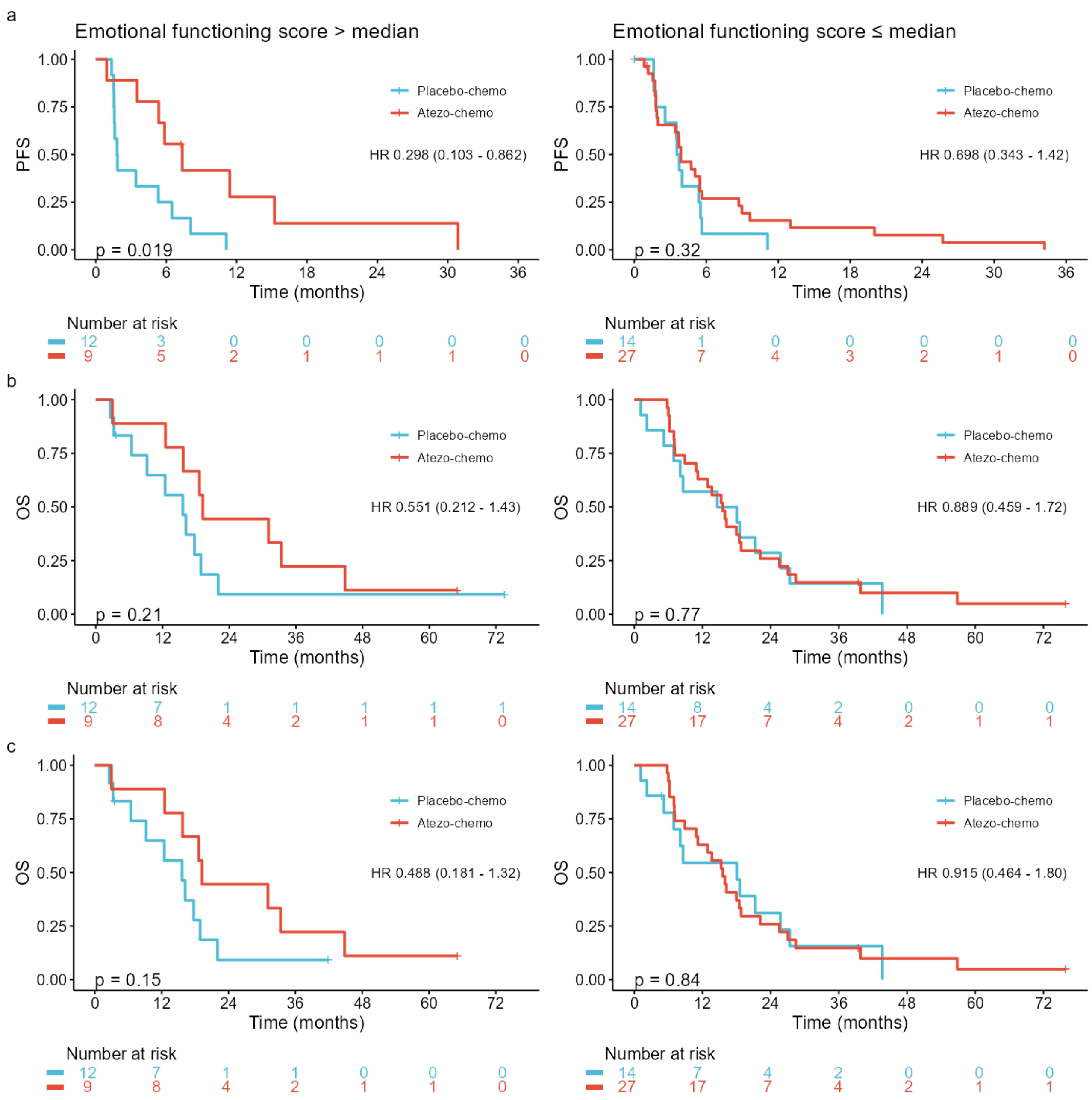

Suppl. Fig. 6: Predictive value of emotional functioning (EF) for the benefit of atezolizumab. Kaplan-Meier plots for PFS (a) and OS (b-c), in the atezo-chemo compared to placebo-chemo arm, in patients with high (>median) or low (≤ median) EF score. Two patients in the placebo-chemo arm started therapy with PD1/PD-L1 checkpoint inhibitors after end-of-treatment in the ALICE trial. (b) OS, without censoring for any post-study therapy. (c) OS, with censoring at start of post-study PD1/PD-L1 checkpoint inhibitor therapy. Median EF score was 83.3. P-values were calculated with log-rank test. HRs were calculated by cox regression analysis.
